# Evaluation of peripheral perfusion index and heart rate variability as early predictors for intradialytic hypotension in critically ill patients

**DOI:** 10.1186/s12871-019-0917-1

**Published:** 2019-12-27

**Authors:** Hanan Mostafa, Mohamed Shaban, Ahmed Hasanin, Hassan Mohamed, Shymaa Fathy, Hossam M. Abdelreheem, Ahmed Lotfy, Ayman Abougabal, Ahmed Mukhtar, Akram El-adawy

**Affiliations:** 1 Department of Anesthesia, Cairo University, Giza, Egypt; 2 Department of Anesthesia and Critical Care Medicine, 01 Elsarayah street, Cairo, 11559 Egypt

**Keywords:** Intradialytic hypotension, Peripheral perfusion index, Heart rate variability

## Abstract

**Background:**

Intradialytic hypotension is a serious complication during renal replacement therapy in critically ill patients. Early prediction of intradialytic hypotension could allow adequate prophylactic measures. In this study we evaluated the ability of peripheral perfusion index (PPI) and heart rate variability (HRV) to predict intradialytic hypotension.

**Methods:**

A prospective observational study included 36 critically ill patients with acute kidney injury during their first session of intermittent hemodialysis. In addition to basic vital signs, PPI was measured using Radical-7 (Masimo) device. Electrical cardiometry (ICON) device was used for measuring cardiac output, systemic vascular resistance, and HRV. All hemodynamic values were recorded at the following time points: 30 min before the hemodialysis session, 15 min before the start of hemodialysis session, every 5 min during the session, and 15 min after the conclusion of the session. The ability of all variables to predict intradialytic hypotension was assessed through area under receiver operating characteristic (AUROC) curve calculation.

**Results:**

Twenty-three patients (64%) had intradialytic hypotension. Patients with pulmonary oedema showed higher risk for development of intradialytic hypotension {Odds ratio (95% CI): 13.75(1.4–136)}. Each of baseline HRV, and baseline PPI showed good predictive properties for intradialytic hypotension {AUROC (95% CI): 0.761(0.59–0.88)}, and 0.721(0.547–0.857)} respectively.

**Conclusions:**

Each of low PPI, low HRV, and the presence of pulmonary oedema are good predictors of intradialytic hypotension.

## Background

Acute kidney injury is common among critically ill patients. Intermittent hemodialysis is one of the commonly used routes for renal replacement therapy [1]. Intradialytic hypotension is a common complication during renal replacement therapy due to volume removal, changes in plasma osmolality, and autonomic dysfunction. In addition to hindering the dialysis session, hypotension impairs successful recovery of kidney function. Intradialytic hypotension might lead to major organ damage, and is sometimes detrimental [2]. Predicting intradialytic hypotension would facilitate initiation of prophylactic measures to decrease its prevalence and severity. Predicting intradialytic hypotension would also impact the decision of the renal replacement towards continuous modality rather than intermittent hemodialysis.

Peripheral Perfusion Index (PPI) is defined as “the ratio of pulsatile blood flow to the non-pulsatile blood flow”. PPI is measured using pulse co-oximetry technology which is characterized by being simple and non-invasive. PPI mirrors the strength of blood flow and quality of perfusion at sensor site, reflecting the global perfusion state of the body [3]. As PPI is generally affected by sympathetic tone, it was a useful early predictor for the need of vasopressors in patients with severe sepsis [4]. PPI had been also reported a useful predictor of hypotension during continuous veno-venous hemofiltration [5]. No data are available for the validity of PPI in early prediction of hypotension during intermittent hemodialysis.

Heart rate variability (HRV) is commonly described as a “new vital sign” which had shown promise in evaluation of autonomic nervous system function [6]. Furthermore, it is considered a useful marker for early risk stratification and prognosis in critically ill patients [6–8].

The aim of this work was to evaluate the ability of PPI and electrical cardiometry-derived HRV to predict hypotension in critically ill patients during intermittent hemodialysis.

## Methods

A prospective observational trial was conducted in Cairo University hospital after Institutional Research Ethics approval (N-91-2018) including a cohort of 36 adult critically ill patients with acute kidney injury (AKI). Written informed consent was obtained from patients or their surrogates before inclusion in the study. We included patients who were scheduled for first session intermittent hemodialysis according to Kidney Disease Improving Global Outcomes (KDIGO) guidelines (pulmonary oedema, uremic complications, hyperkalemia not responding to other measures and intractable acidosis) [9]. All enrolled patients were classified as KDIGO stage III [9].

We excluded patients with pre-existing end-stage renal disease, patients with severe vascular disease compromising measurements of PPI, and patients with major burns which precluded the application of electrical cardiometry electrodes.

### Hemodialysis

Acute kidney injury was diagnosed if the patient showed any of the following criteria: 1- Increase in serum creatinine by at least 0.3 mg/dL within 48 h. 2- Increase in serum creatinine to 1.5 times baseline. 3- Urine volume of less than 0.5 mL/kg per hour for 6 h. Hemodialysis was decided by the attending nephrologist for volume overload, severe electrolyte disturbance (Acidosis, hyperkalemia) or severe uremia [9].

Parameters of dialysis session were determined by the attending nephrologist with pump rate of 200–250 ml/min, session time not exceeding 3 h, and maximum ultrafiltration rate of 1 L/hour, Hemodialysis sessions were performed using hemodialysis machine (Gambro AK96), and 1.7 m^2^ biocompatible filters.

### Monitoring

Standard monitors were applied for all patients during the session of hemodialysis. Non-invasive arterial blood pressure (systolic, diastolic and mean) monitor was measured every 5 min. Electrocardiogram (ECG) and pulse oximetry were applied continuously. Central venous pressure (CVP) was measured via a right internal jugular central venous catheter.

PPI was measured using Masimo SET Radical- 7 device (Masimo Corp., Irvine, Calif., USA). The adhesive sensor was attached onto the index finger (Masimo SET® LNCS Adtx, adult sensor).

Electrical cardiometry ICON device (Osyka Medical, Berlin Germany) was used to measure advanced hemodynamic variables (cardiac output, systemic vascular resistance, and HRV). HRV analysis by the electrical cardiometry was determined using time-domain analysis, specifically standard deviation of the normal-to-normal R-R interval [8].

Hypotensive episode was diagnosed as 20% reduction of mean blood pressure from the baseline value which required either initiation or increased rate of norepinephrine infusion. Patients were categorized into 2 groups. Hypotensive group and stable group. Hypotensive group included any patient who had one or more hypotensive episodes.

### Outcomes

Our primary outcome was to detect the predictive ability of PPI for intradialytic hypotension. Secondary outcomes included non-invasive blood pressure, heart rate, cardiac output, systemic vascular resistance, CVP, and HRV. All hemodynamic values were recorded at the following time points: 30 min before the hemodialysis session, 15 min before the start of hemodialysis session, every 5 min during the session, and 15 min after the conclusion of the session.

### Statistical analysis

Our primary outcome was the area under receiver operating characteristic (AUROC) curve for PPI in prediction of intradialytic hypotension. In a previous study, the AUROC for PPI for prediction of hypotension during continuous hemodialysis was 0.8 [5]. Thus, we calculated our sample size using MedCalc version 12.1.4.0 (MedCalc Software bvba, Mariakerke, Belgium) to detect AUROC of 0.8 with null hypothesis of 0.5. The calculated minimum number of patients to have a study power of 80% and alpha error of 0.05 was 26 patients with at least 13 positive and 13 negative cases.

Statistical analysis was performed using SPSS 15 (Chicago, IL). Categorical data were presented as frequency (%); continuous data were checked for normal distribution by Kolmogorov-Smirnov test. Normally distributed continuous data were presented as mean ± SD, and skewed data were presented as median (quartiles). Patients were classified into hypotensive patients and stable patients; Fisher’s exact test was used to compare frequencies between the two groups. Unpaired t-test and Mann-Whitney test were used to compare the means for continuous data as appropriate.

To compare the performance of different variables in predicting intradialytic hypotension, receiver operating characteristic (ROC) curves were constructed and the AUROC curve was calculated for each variable. MedCalc software generated values with the highest sensitivity and specificity (Youden index). The AUROC curves were compared using a Hanley-McNeil test. The level of significance was set at *P* < 0.05 for two-tailed tests.

## Results

Forty-one patients were screened for eligibility. Five patients were excluded during the hemodialysis session due to weak device signals; 36 patients were included in the study, and all of them were available for final analysis. Causes of ICU admission included septic shock (15 patients [42%]), pulmonary edema (6 patients [17%]), eclampsia (1 patient [3%]), diabetic ketoacidosis (2 patients [6%]), and disturbed conscious level due to head trauma (8 patients [22%]) or non-traumatic intracranial hemorrhage (4 patients [10%]). Four (11%) patients were mechanically ventilated and four (11%) patients were on norepinephrine infusion before starting the hemodialysis session. Twenty-three (64%) patients had intradialytic hypotension. Demographic data and patient chronic comorbidities were comparable between hypotensive group and stable group; whilst, APACHE II score was higher in the hypotensive group compared to stable group (Table 1). Heart rate, cardiac output, and systemic vascular resistance were comparable between both groups during the dialysis session. Higher baseline systolic blood pressure was associated with lower risk of intradialytic hypotension (odds ratio [95%]: 0.95 [0.89–0.98]). The incidence of intradialytic hypotension was higher in patients admitted to dialysis due to pulmonary edema (odds ratio [95% confidence interval]: 13.75[1.4–136]). (Table 1).

**Table 1:** Demographic data and patient characteristics. Data are presented as mean ± standard deviation, median (quartiles), and frequency (%)

	Hypotensive group (*n* = 23)	Stable Group (*n* = 13)	*P* value
Age (years)	49 ± 13	55 ± 9	0.15
Male gender	20 (87%)	8 (62%)	0.1
APACHE II	26 ± 6	21 ± 6	0.02
Diabetes	12(52%)	10(77%)	0.18
Chronic hypertension	16(70%)	10(77%)	0.72
Ischemic heart disease	3(13%)	3(23%)	0.4
Chronic liver disease	2(9%)	3(23%)	0.3
Pulmonary edema	1(4%)	5(39%)	0.02
Fluid removed during session	1615 ± 650	1543 ± 562	0.73
Baseline SBP (mmHg)	120(113,127)	148(122,155)	0.001
Baseline heart rate (bpm)	96 ± 18	92 ± 15	0.51
Baseline CVP (cmH_2_O)	16 ± 6	15 ± 5	0.55
Baseline cardiac output (L/minute)	9.3 ± 3	9.7 ± 2.4	0.67
Baseline systemic vascular resistance (dynes.sec.cm^− 5^)	806(686,1252)	729(631,980)	0.149

*APACHE* Acute physiology and chronic health evaluation, *CVP* central venous pressure, *SBP* systolic blood pressure

**Fig. 1:**
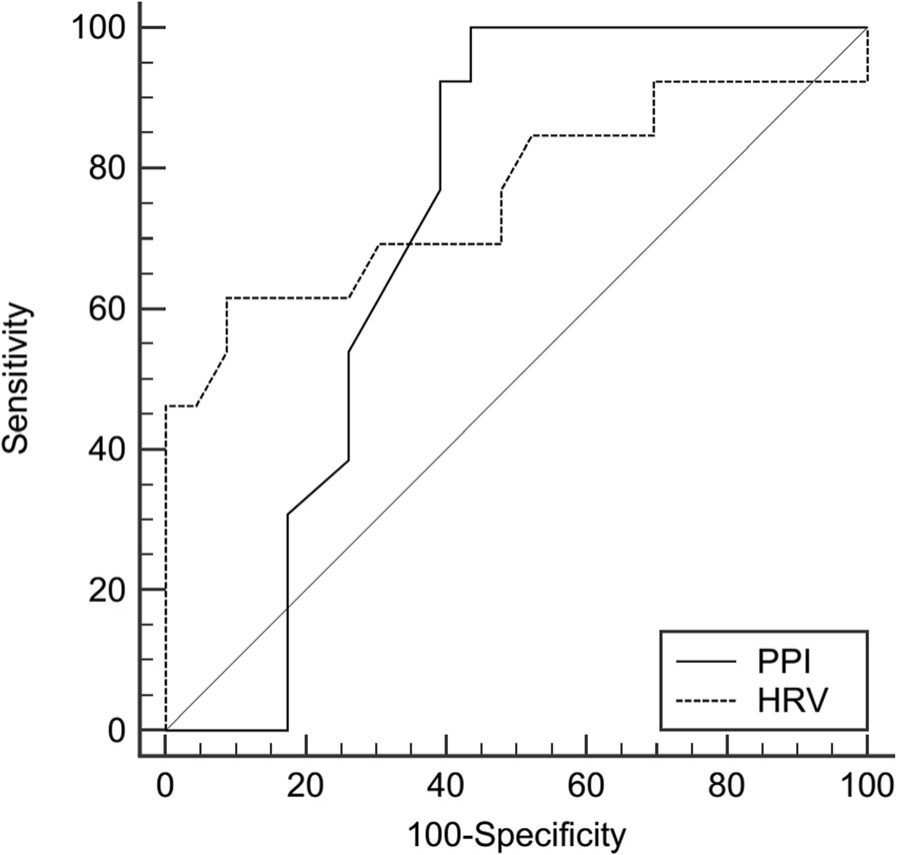
Receiver operating characteristic curves for different variables to detect intradialytic hypotension. HRV: heart rate variability, PPI: peripheral perfusion index

Baseline HRV and baseline PPI showed good predictive ability of intradialytic hypotension {AUROC (95% CI): 0.761(0.59–0.88), cut-off value ≤24}, and {AUROC (95% CI): 0.721(0.547–0.857), cut-off value ≤1.8} respectively. PPI was superior in terms of sensitivity and negative predictive value (NPV) (100 and 100%); whilst, HRV was superior in terms of specificity and positive predictive value (PPV) (91 and 92%). (Table 2) (Fig. 1).

**Table 2:** Predictive properties for perfusion index and electrical cardiometry measures for hypotension

	AUROC	95% CI	Sensitivity	Specificity	PPV	NPV	Cut-off value
PPI	0.721	0.547–0.857	100%	56%	80%	100%	≤1.8
HRV	0.761	0.59–0.887	61%	91%	92%	56%	≤24

*HRV* heart rate variability, *PPI* peripheral perfusion index

## Discussion

We evaluated two simple, non-invasive, hemodynamic variables, namely PPI and HRV, for early prediction of intradialytic hypotension. Both variables showed good ability for early detection of high-risk patients. HRV showed better specificity; whilst, PPI showed good sensitivity.

In our results PPI showed good predictive ability for intradialytic hypotension. PPI is considered an objective measure for peripheral perfusion which changes according to the change in the pulsatile blood flow. Thus, PPI decreases with sympathetic stimulation and vasoconstriction [10] and increases with sympathetic block and vasodilatation [11, 12]. The association of low baseline PPI with intradialytic hypotension is most probably because these patients have a high sympathetic tone and may be unable to compensate for ultrafiltration induced hypovolemia with further vasoconstriction making them more vulnerable to intradialytic hypotension.

In line with our findings, Klijn et al. [5] reported good predictive ability for PPI for detection of hypotension due to fluid withdrawal during continuous veno-venous hemofiltration. Klijn et al. reported lower different cutoff value than ours (0.82 versus 1.8). Klijn and colleagues included patients on continuous hemodialysis who usually need vasopressor support (61% of their patients were on vasopressors before initiation of continuous hemodialysis). The use of vasopressors decreases the PPI and would be responsible for the lower cutoff value in Klijn et al. patients compared to our patients.

Low HRV showed good predictive value for intradialytic hypotension in our patients. HRV represents the balance between sympathetic and parasympathetic nervous systems. Thus, HRV had been considered a marker of two conditions, cardiovascular well-being and acute illness [8]. The tolerance to fluid removal during hemodialysis depends on compensatory cardiovascular reflexes which need intact autonomic nervous system; thus, autonomic dysfunction had been considered as an important risk factor for intradialytic hypotension [13, 14] . Autonomic nervous system can be overwhelmed with the pathological process in critically ill patients with the sympathetic system exerting its maximum effort to maintain blood pressure and peripheral perfusion. Low HRV can correspond to exhausted autonomic system that can no longer respond to further stresses leaving the patient defenseless in front of new stressful events. Autonomic dysregulation has been deemed to be a contributing factor in the pathogenesis of intradialytic hypotension [15, 16]. Supporting our findings, Rubinger et al. had reported that low HRV was associated with intradialytic hypotension in chronic hemodialysis patients [17]. Low HRV was also predictive for perioperative hypotension in various types of surgery [8].

In our cohort, a strong association was reported between volume overload (pulmonary oedema) and intradialytic hypotension with Odds ratio (95% confidence interval) of 13.75(1.4–136). Volume overload had been considered as a biomarker for the severity of critical illness [18]. Fluid overload in critically ill patients on renal replacement therapy was associated with increased mortality rate [19] [20] and higher need of vasopressor therapy during intensive care unit stay [19]. Thus, we suggest that this subgroup of patients with pulmonary oedema had more severe illness and profound physiological derangement.

Our study has the advantage of using simple non-invasive variables. Furthermore, we evaluated patients during the usual route for renal replacement therapy which is intermittent hemodialysis. Intradialytic hypotension is a common and serious complication during hemodialysis. Early detection of high-risk patients for intradialytic hypotension would help to decrease their risk through early initiation of vasopressors. High filtration rate and ultrafiltration volume are important factors which contribute in intradialytic hypotension [21]; Hence, minimizing ultrafiltration and decreasing the rate of fluid removal could help in avoiding hypotension in high-risk patients. Other routes for avoiding intradialytic hypotension include minimizing reductions in osmolarity, and finally, shifting to continuous hemodialysis. This study had some limitations: 1- It is a single center study. 2- We included a mixed cohort of critically ill patients; further subgroup analyses in future studies might modify our cutoff values. 3- The study was not powered enough to perform multivariate analysis. 4- Our findings are reported during certain filtration parameters and needed to be confirmed in future studies in patients whom ultrafiltration is performed through different rates and protocols.

## Conclusions

Low PPI, low HRV, and the presence of pulmonary oedema are useful predictors of intradialytic hypotension.

## Data Availability

The data that support the findings of this study are available from Cairo university hospitals; however, they are not publicly available. Data are however available from the authors upon reasonable request after permission of Cairo University.

## Abbreviations

Age: Chronic Health Evaluation
APACHE: Acute Physiology
AUROC: Area under receiver operating characteristic
CVP: Central venous pressure
ECG: Electrocardiogram
HRV: Heart rate variability
KDIGO: Kidney Disease Improving Global Outcomes
NPV: Negative predictive value
PPI: Peripheral perfusion index
PPV: Positive predictive value
